# Near full-lenght genome characterization of a newly derived unique recombinant form AG HIV-1 circulating in Siberia

**DOI:** 10.1186/1742-4690-9-S1-P28

**Published:** 2012-05-25

**Authors:** Pavel Borisovich Baryshev, Natalya Matveevna Gashnikova, Vladislav Viktorovich Bogachev

**Affiliations:** 1 State Research Center of Virology and Biotechnology VECTOR, Koltsovo, Russia

## Background

Genotyping of HIV-1 variants isolated from patients of Siberian region (Russia) in 2006-2010 revealed a sharp increase of 02_AG recombinant variant proportion among HIV-1 genetic variants, circulating in the Siberia, from 2% in 2006 to 52% in 2010. The majority of HIV 02_AG variants isolated in Siberia form a separate branch of phylogenetic tree.

The objective of this analysis was to study structure of the near full-length genome sequence of HIV-1 strains recombinant form AG rapidly spreading in Siberia.

## Material and methods

Sample 10.RU.6637 was collected in 2009 from a 28-year-old man, infected in 2008 through heterosexual contact. Three regions of the viral genome were independently amplified using nested PCR from the cDNA to contain a nearly full-length genome of HIV-1. Sequencing of all amplicons was performed by using cycle sequencing and dye termination on an automated sequencer, DNA sequences were assembled using Sequencher software. Jumping profile hidden Markov model (jpHMM) program was used to analyze the subtype assignment of all sequences retrieved. The software Simplot v3.5.1 was initially used to perform bootscanning analyses of a query sequence against a set of other sequences. Phylogenetic trees were constructed with the program PhyML v.3.0 using a maximum likelihood approach.

## Results

Sequence 10.RU.6637 HIV-1 from Siberia was submitted to Genbank under accession number JN230353. Points of recombination were determined and uniqueness of 10.RU.6637 HIV-1 genome structure was shown through in-depth genetic analysis. Recombinant breakpoint analysis of 10.RU.6637 HIV-1 genome sequence revealed that it was recombinant form between CRF 02_AG and sub-subtype A1. We will designate this variant 02_AG/IDU A HIV-1, to reflect its close relationship to the CRF 02_AG and A1 (IDU A) subtype from Russia.

## Conclusion

The majority of HIV 02_AG variants circulating in Siberia represented of a newly derived unique recombinant form 02_AG/IDU A HIV-1. Wider spread of HIV-1 URF02_AG/IDU A is possible in the territory of Russia in the coming years.

